# Stool fatty acid soaps, stool consistency and gastrointestinal tolerance in term infants fed infant formulas containing high *sn*-2 palmitate with or without oligofructose: a double-blind, randomized clinical trial

**DOI:** 10.1186/1475-2891-13-105

**Published:** 2014-11-05

**Authors:** Joyce Nowacki, Hung-Chang Lee, Reyin Lien, Shao-Wen Cheng, Sung-Tse Li, Manjiang Yao, Robert Northington, Ingrid Jan, Gisella Mutungi

**Affiliations:** Nestlé Nutrition, 3000 Horizon Drive, King of Prussia, PA 19406 USA; Mackay Memorial Hospital, Taipei City, Taiwan; Chang Gung Medical Foundation- LinKuo Branch, Tao-Yuan County, Taiwan; Chang Gung Medical Foundation - Taipei Branch, Taipei City, Taiwan; Mackay Memorial Hospital, Hsinchu Branch, Hsinchu City, Taiwan; Graduate Institute of Business Administration, College of Management, Fu Jen Catholic University, New Taipei City, Taiwan; Nestlé Taiwan Limited, Taipei City, Taiwan

**Keywords:** Oligofructose, *sn*-2 palmitate, Palmitate soaps, Fatty acid soaps, Stool consistency, Term infant formula

## Abstract

**Background:**

Formula-fed (FF) infants often have harder stools and higher stool concentrations of fatty acid soaps compared to breastfed infants. Feeding high *sn*-2 palmitate or the prebiotic oligofructose (OF) may soften stools, reduce stool soaps, and decrease fecal calcium loss.

**Methods:**

We investigated the effect of high *sn*-2 palmitate alone and in combination with OF on stool palmitate soap, total soap and calcium concentrations, stool consistency, gastrointestinal (GI) tolerance, anthropometrics, and hydration in FF infants. This double-blind trial randomized 165 healthy term infants 25–45 days old to receive Control formula (n = 54), formula containing high *sn*-2 palmitate (*sn*-2; n = 56), or formula containing high *sn*-2 palmitate plus 3 g/L OF (*sn*-2+OF; n = 55). A non-randomized human milk (HM)-fed group was also included (n = 55). The primary endpoint, stool composition, was determined after 28 days of feeding, and was assessed using ANOVA accompanied by pairwise comparisons. Stool consistency, GI tolerance and hydration were assessed at baseline, day 14 (GI tolerance only) and day 28.

**Results:**

Infants fed *sn*-2 had lower stool palmitate soaps compared to Control (*P* =0.0028); while those fed *sn*-2+OF had reduced stool palmitate soaps compared to both Control and *sn*-2 (both *P* <0.0001). Stool total soaps and calcium were lower in the *sn*-2+OF group than either Control (*P* <0.0001) or *sn*-2 (*P* <0.0001). The HM-fed group had lower stool palmitate soaps, total soaps and calcium (*P* <0.0001 for each comparison) than all FF groups. The stool consistency score of the *sn*-2+OF group was lower than Control and *sn*-2 (*P* <0.0001), but higher than the HM-fed group (*P* <0.0001). GI tolerance was similar and anthropometric z-scores were <0.2 SD from the WHO growth standards in all groups, while urinary hydration markers were within normal range for all FF infants.

**Conclusions:**

Increasing *sn*-2 palmitate in infant formula reduces stool palmitate soaps. A combination of high *sn*-2 palmitate and OF reduces stool palmitate soaps, total soaps and calcium, while promoting softer stools.

**Trial registration:**

This study was registered on http://www.clinicaltrials.gov: number NCT02031003.

**Electronic supplementary material:**

The online version of this article (doi:10.1186/1475-2891-13-105) contains supplementary material, which is available to authorized users.

## Background

Human milk (HM) is exceptionally complex containing numerous components that function in a synergistic manner. Nutritional components designed to improve infant formula (IF) are often added and evaluated in a step-wise process. However, IF ingredients may interact with each other, and consequently, the evaluation of isolated ingredients is insufficient in assessing these complex relationships. One example of such interactions involves the digestion of triglycerides and the subsequent absorption of fatty acids and calcium [1], processes which are dependent on both lipid structure and intestinal environment (such as colonic bacterial populations and pH).

Fatty acids in HM and IF triglycerides are esterified to the *sn*-1, *sn*-2 and *sn*-3 positions of the glycerol backbone [2]. Palmitic acid (PA) comprises approximately 20 percent of total HM fatty acids [3], of which approximately two thirds is esterified to the triglyceride *sn*-2 position [2]. In contrast, greater than 80 percent of PA in IF fat blends is esterified to the *sn*-1 and *sn*-3 positions [4]. During digestion, the *sn*-1 and *sn*-3 fatty acids are hydrolyzed by pancreatic lipase. The selective activity of pancreatic lipase on the *sn*-1 and *sn*-3 positions of the triglyceride generates two free fatty acids and a corresponding *sn*-2 monoglyceride. This *sn*-2 monoglyceride is readily absorbed regardless of the structure of its esterified fatty acid [1]. The absorption characteristics of the free fatty acids are dependent on their structure. Of the free fatty acids, saturated fatty acids are less readily absorbed than unsaturated fatty acids and have the potential to form insoluble fatty acid soaps, which can result in the loss of both calcium and energy in the feces. Compared to the hydrolysis of HM triglycerides, hydrolysis of IF triglycerides generates more free PA with the potential to bind calcium and form PA soaps [5]. Since any reduction in stool soap formation is likely to result in reduced fecal loss of both energy and calcium, the development of a structured triglyceride (closer to HM with high *sn*-2 PA) and its addition to IF will be of benefit to FF infants*.*

Other means of reducing fecal calcium loss are available and the activities of prebiotics are ideally suited to this purpose. OF is a fructan oligosaccharide derived from enzymatic hydrolysis of chicory root inulin and consists of 2 – 8 fructose residues. It functions as a soluble dietary fiber with prebiotic activities in that it is not digested in the small intestine, but is fermented in the colon, stimulating the growth of colonic populations of beneficial bacteria [6] and lowering pH [7], which may increase the solubility of calcium in the colon and subsequently increase calcium absorption [8, 9].

Stools are harder in FF infants than in infants fed breast milk [5, 10] and may be difficult to pass; therefore, stool consistency is often a concern of parents of FF infants [11]. Stool consistency is influenced by numerous factors, including the presence of PA-calcium soaps [5] and colonic fermentation of non-digestible oligosaccharides [6]. Thus the addition of both high *sn*-2 PA triglyceride and prebiotic to IF may improve stool consistency.

The objective of the present study was to evaluate the effects of feeding IFs containing either high *sn*-2 palmitate or high *sn*-2 palmitate with the addition of 3 g/L OF compared to a Control formula. A HM-fed reference group was also included. We determined the stool concentrations of fatty acid soaps and calcium as well as stool consistency and frequency. Since runny or watery stool may suggest altered water balance, urinary markers of hydration were also measured.

## Methods

### Study population

In this multi-center, randomized, controlled, double-blind, parallel-group study, healthy, term infants born at 37–42 weeks gestation were enrolled at 25–45 days of age and randomized to receive one of three IFs. In addition, a non-randomized reference group exclusively receiving HM was included. FF infants had to be exclusively consuming and tolerating a cow’s milk IF and the parent/legal guardian must have previously made a decision to continue to exclusively formula feed. The purpose of the study and the procedures, benefits and risks involved in study participation were explained to the parents/legal guardians of all participating infants before written informed consent was obtained. Prior to implementing any study procedures, each subject’s parent/legal guardian voluntarily signed and dated the informed consent.

Exclusion criteria included: a history of siblings with documented cow’s milk protein intolerance/allergy; major congenital malformations; suspected or documented systemic or congenital infections (e.g., human immunodeficiency virus, cytomegalovirus); infants who received any medication (s) which were known or suspected to affect fat digestion, absorption and/or metabolism (e.g., pancreatic enzymes), any vitamin and/or mineral supplements containing calcium, suppositories, bismuth containing medications, herbal supplements, or medications that could neutralize or suppress gastric acid secretion; or conditions requiring infant feedings other than those specified in the protocol.

All infants were fed ad libitum for a period of four weeks and were followed for a period of two weeks after last study feeding. Parents and infants participated in clinic visits at baseline, day 14 and day 28 with telephone interviews at days 7, 21 and 42. At baseline, day 14 and day 28, anthropometric data were collected and parents completed the Infant Gastrointestinal Symptom Questionnaire (IGSQ) [12] to assess GI tolerance. For three consecutive days immediately before the day 14 and day 28 clinic visits, parents completed a stool consistency and frequency diary. Prior to the day 28 clinic visit, parents collected stool for analysis of fatty acid soaps and mineral content. Adverse event information was collected at all clinic visits and during all telephone calls.

The study was conducted between 13 September, 2010 and 20 December, 2011 at four hospitals in Taiwan: Mackay Memorial Hospital, Taipei City; Mackay Memorial Hospital, Hsinchu Branch; Chang Gung Medical Foundation, Taipei Branch; and Chang Gung Medical Foundation, Linkuo Branch. It was performed in accordance with the guidelines of the Declaration of Helsinki in conformance with International Conference on Harmonization, Good Clinical Practice and the criteria described in 21 Code of Federal Regulations 312.120 (Foreign Clinical Studies Not Conducted Under an Investigational New Drug Application). The study protocol, protocol amendments, and informed consent form were reviewed and approved by each hospital’s Institutional Ethical Review Board according to ICH-GCP guidelines.

### Randomization

At study entry, FF infants were randomly assigned in a 2:2:2 ratio (block size of 6) utilizing the validated randomization software, TriaLine system (INC Research), to receive one of three study formulas. Site personnel were provided with a subject randomization number and a package number to assign to each subject. Assigned formula packages were dispensed on study days 1 and 14. Study formula was packaged identically except for the unique 5 digit package identification number printed on the label, so that the investigator, study staff, participants, and care providers were blinded to the allocated treatment.

### Composition of study formula

The three study formulas were of similar composition, with the exception of the fat blend source and the presence of OF: 1.) Control formula, whey-predominant and enriched in alpha-lactalbumin with a 100% vegetable fat blend; 2.) *sn*-2 formula, Control formula with a fat blend containing 60% Control vegetable oil and 40% high *sn*-2 palmitate vegetable oil; and 3.) *sn*-2+OF formula, the *sn*-2 formula supplemented with 3.0 g/L OF (Orafti® P95, BENEO-ORAFTI, Tienen, Belgium). Table 1 provides fatty acid profiles, positional distribution of PA and selected nutrient concentrations of each study formula.

**Table 1:** **Selected nutrient composition, fatty acid profiles and positional distribution of PA of the study formulas**

	Formula group		
Nutrient/Ingredient	Control	***sn*** -2	***sn*** -2+OF
Energy, kcal/L	661	662	678
Protein, g/L	14	15	15
Total Fat, g/L	36	37	37
Carbohydrate, g/L	69	69	72
Calcium, mg/L	411	423	431
Oligofructose, g/L	-	-	3
Fatty acids, weight % of TFAs			
C12:0, lauric	7.1	6.8	6.9
C14:0, myristic	4.1	3.4	3.5
C16:0, palmitic	21.1	22.5	22.5
C18:0, stearic	3.9	3.7	3.6
C18:1, oleic	39.4	40.1	40.0
C18:2, linoleic	18.5	17.2	17.1
18:3, linolenic	1.4	1.5	1.5
C20:4, arachidonic	0.3	0.4	0.4
C22:6, docosahexaenoic	0.2	0.2	0.2
Percent of PA (C16:0) in the *sn*-2 position	12.6	38.9	40.8

Control = bovine milk-based, whey-predominant, alpha-lactalbumin-enriched term infant formula with 100% vegetable fat blend; PA = palmitic acid; *sn*-2 = high *sn*-2 palmitate formula (Control formula modified to contain 60% vegetable fat blend and 40% high *sn*-2 palmitate fat blend); *sn*-2+OF = high *sn*-2 palmitate formula supplemented with oligofructose at 3.0 g/L; TFAs = total fatty acids.

### Stool characteristics and biochemical composition

Information related to stool characteristics (stool consistency and frequency) was collected using a three-day stool diary that parents completed immediately prior to the day 14 and day 28 visits. Each stool that was passed during the three-day period was evaluated by the parent/caregiver according to a validated 5-point scale (1 = Watery, 2 = Runny, 3 = Mushy Soft, 4 = Formed, 5 = Hard) [5, 10]. The number of bowel movements per day was also recorded. An average stool consistency score and the average number of stools per day were determined for each three-day period.

Stool samples were collected at home beginning on day 22 (±3 days). The collection involved use of diapers fitted with a strip of Tegaderm tape (to facilitate stool retention in the diaper) and placement of each freshly passed stool in an amber plastic bag. The bag was weighed using a portable gram scale and stored within a stool collection container in a home freezer until ≈ 30 g had been collected. Frozen stools were transported from home to study center in an insulated bag, then transported on dry ice to Covance Laboratories, Inc., Madison, Wisconsin, USA for analysis. The analytical laboratory determined fatty acid soap content utilizing a modified method of Quinlan et al. [5]. Briefly, samples were thawed, wet weight determined, then lyophilized and dry weight determined. Neutral lipids were extracted from 0.5-1.0 g freeze-dried sample of stool by reflux with petroleum ether. The remaining sample was acidified utilizing acetic acid in order to convert fatty acid soaps to free fatty acids, which were then isolated by reflux with petroleum ether. Free fatty acids from each extract were spiked with internal standards and isolated by use of an anhydrous alkaline exchange resin. Fatty acid methyl esters were synthesized by use of hydrochloric acid and methanol followed by capillary gas chromatography analysis. Total fatty acid soaps were derived from the sum of all measured individual fatty acid soaps. All fatty acid soap data are reported as mg per g of dry weight stool. Percent stool moisture was determined by measuring the stool sample wet weight (g) and subtracting the dry stool weight (g) after lyophilization, divided by the wet stool weight and then multiplied by 100. Stool solids were determined by subtracting % stool moisture from 100%. Stool calcium content was determined by ICPL (Inductively Coupled Plasma) Emission Spectrometry utilizing the AOAC International Official Methods of Analysis protocol [13]. The stool sample was dried, precharred, and ashed twice. It was then treated with hydrochloric acid, dried, and added to a 5% hydrochloric acid solution. The amount of calcium in the solution was determined on the inductively coupled plasma emission spectrometer by comparing emissions to those of the standard solution.

### Hydration assessment

Due to the possibility of looser, more watery stools in the infants receiving *sn*-2 and *sn*-2+OF formulas, markers of hydration, urine osmolality and urine specific gravity were measured. Urine samples were collected at baseline and day 28 clinic visits using a Medline pediatric urine collection bag. Urine was transferred from the urine collection bag to two labeled Vacutainer® tubes for transport (one without preservative for urine osmolality, and one with preservative tablet for urine specific gravity). Samples were shipped on day of collection at ambient temperature to Covance Central Laboratory Services, Singapore. Temperature was maintained during shipment using Gel-Pak thermal insulators. Once samples were received at Covance Singapore, they were frozen and shipped to Covance Central Laboratory Services, Indianapolis, Indiana, USA for analysis of urine osmolality and urine specific gravity using standard laboratory methods.

### GI tolerance

The GI Symptom Burden Index Score [12] was used to assess parent-reported GI tolerance. This index score is based on a subset of 13 questions included in the IGSQ. The IGSQ is a standardized, validated, interviewer-assisted questionnaire containing 21 questions across 5 GI categories: stooling, vomiting, crying, fussiness, and flatulence, allowing parents to report the frequency and intensity of their infant’s GI symptoms from the previous 7 days. For determination of the index score, the responses to 13 of the questions are summed to produce a single score, which is a measure of the total GI symptom burden. The potential range for the GI Symptom Burden Index Score is 13 to 65; a score of 13 indicates low GI burden, while a score of 65 indicates high GI burden. Parent-reported GI tolerance was descriptively summarized for each formula group and the HM group at baseline, day 14, and day 28 clinic visits.

### Adverse events

An adverse event (AE) was defined as any untoward, undesired, or unplanned event in the form of signs, symptoms, disease, or laboratory or physiological observations that occurred in an infant from the time of informed consent signing until approximately 2 weeks after last assigned study feeding. The event did not necessarily need to be causally related to the study formula or study procedures. A serious AE (SAE) was defined as an AE that resulted in death, was life-threatening, required inpatient hospitalization or prolongation of an existing hospitalization, or resulted in a persistent or significant disability or incapacity, resulted in cancer, was a congenital anomaly or birth defect, or an important medical event. AEs were recorded by the hospital staff during clinic visits and telephone contacts with parents throughout the study and 14 days after completion of the feeding portion of the study. Particular attention was paid to GI AEs.

### Anthropometry

Infants were weighed on an electronic infant scale (Seca 374, Hamburg, Germany) without a diaper; weight was recorded to the nearest gram. A pediatric length board (NWB LB 35-70-X; Ellard Instrumentation, Washington, DC, USA) was used to measure recumbent length to the nearest 0.1 cm; head circumference was measured with a pediatric tape measure (Seca 212, Hamburg, Germany) to the nearest 0.1 cm. All measures were taken twice and the mean was calculated.

### Statistical methods

Sample size was based on power calculations for the primary outcome (stool fatty acid soaps) and a key secondary outcome (stool consistency). Based on these calculations, 23 infants per group were required for 90% power to detect a mean (SD) between-group difference of 7% (7%) in fatty acid soaps at α = 0.05; and 40 infants per group were required for 90% power to detect a mean (SD) between-group difference of 0.3 (0.4) points in the five-point stool consistency scale at α = 0.05 (Details on these calculations are provided in the Additional file 1). Based on the higher number, a minimum of 40 infants per group was needed for adequate power. To account for dropouts, enrollment of 55 infants per group was required.

Comparison of the three FF groups was of primary interest. There was no overall analysis for differences among all four feeding groups, primarily because the HM-fed group was included in the study as a reference group. Therefore, it was considered appropriate to perform the primary comparisons of the FF groups independent of the HM-fed group. ANOVA was used to assess mean differences in stool composition across the three FF groups. Accompanying each overall analysis was a series of pairwise comparisons conducted to test for differences between each pair of FF groups using the pooled error term. Additionally, a series of independent t-tests to compare each of the FF groups separately with the HM-fed group was conducted.

For analysis of stool consistency and stool frequency data, a mixed model repeated measures ANCOVA was used to compare the three FF groups. In addition, separate repeated measures analyses were done for pairwise comparisons between the HM-fed group and each of the three FF groups. All statistical tests were 2-sided and performed at α = 0.05. A step-wise process was used to control for multiple comparisons (Details on this process are provided in Additional file 1).

## Results

### Study population

**Figure 1:**
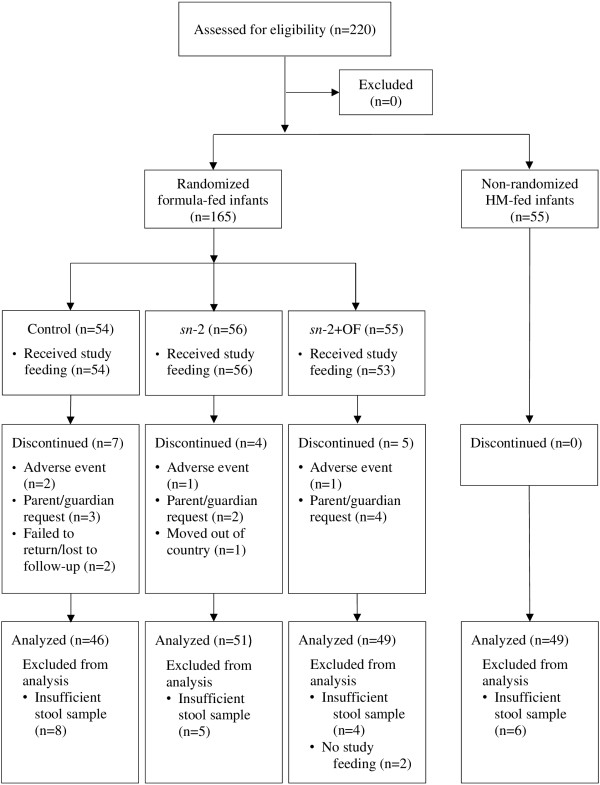
**Study flow diagram.** Control = bovine milk-based, whey-predominant, alpha-lactalbumin-enriched term infant formula with 100% vegetable fat blend; HM = human milk; *sn*-2 = high *sn*-2 palmitate formula (Control formula modified to contain 60% vegetable fat blend and 40% high *sn*-2 palmitate fat blend); *sn*-2+OF = high *sn*-2 palmitate formula supplemented with oligofructose at 3.0 g/L.

A total of 165 FF infants (randomized) and 65 HM-fed infants (non-randomized) were enrolled and participated in the study (Figure 1) between September, 2010 and December, 2011. Infants in the randomized groups were similar in terms of gestational age, age at enrollment, birth order, gender and race (Table 2). Household characteristics in terms of maternal age, parity, mode of delivery, parents’ number of school years completed, marital status, and infant feeding history, were similar for the three FF groups.

**Table 2:** **Baseline demographic characteristics of the study population**

	Feeding group			
	Control	***sn*** -2	***sn*** -2+OF	HM
	(n =46)	(n =51)	(n =49)	(n =49)
Infant characteristics				
Age at enrollment, days^*1*,*2*^	34.5 (3.1)	35.2 (4.2)	35.2 (3.4)	34.8 (3.9)
Gestational age, weeks^*1,2*^	38.8 (1.2)	38.9 (1.2)	38.8 (1.1)	39.1 (1.0)
Gender, % male^*3,4*^	52	53	59	59
Birth order, % first born^*3,4*^	46	51	41	47
Parental characteristics				
Maternal age, years^*1,2*^	30.7 (5.9)	31.4 (5.5)	31.6 (5.7)	30.5 (4.5)
Marital status, % married^*3,4*^	96	86	96	100
Mother’s number of school years completed^*1,2*^	13.7 (2.1)	14.1 (2.7)	13.8 (1.9)	14.6 (2.9)
Father’s number of school years completed^*1,2*^	13.8 (3.2)	14.1 (2.6)	13.3 (2.7)	15.1 (2.8)

^*1*^Mean (SD).

^*2*^No significant differences were observed based on overall and pairwise comparisons across the formula groups and independent 2 sample t-tests comparing the FF groups to the HM group for infant age, gestational age, maternal age and years of school completed by the mother and father.

^*3*^Percentages are based on the number of subjects in each feeding group.

^*4*^No significant differences were observed based on overall and pairwise comparisons across the formula groups and HM based on overall and pairwise comparisons using Fisher’s exact test for infant gender, infant birth order, and mother’s marital status.

Control = bovine milk-based, whey-predominant, alpha-lactalbumin-enriched term infant formula with 100% vegetable fat blend; HM = human milk; *sn*-2 = high *sn*-2 palmitate formula (Control formula modified to contain 60% vegetable fat blend and 40% high *sn*-2 palmitate fat blend); *sn*-2+OF = high *sn*-2 palmitate formula supplemented with oligofructose at 3.0 g/L.

### Stool lipids and calcium

**Figure 2:**
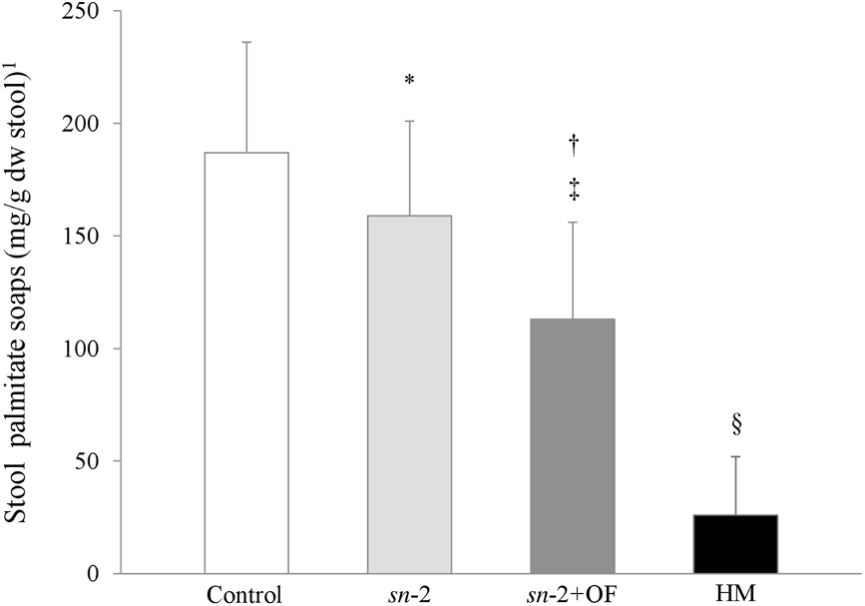
**Stool palmitate soaps (mg/g dry weight stool) at Day 28 according to feeding group.** ^*1*^Values are means ± SD. The overall formula-fed groups were analyzed by ANOVA followed by pairwise comparisons. The HM group was compared to each formula group using independent t-tests. Means (± SD) significantly different from Control: ^*^ *P* =0.0028, ^†^ *P* <0.0001; significantly different from *sn*-2: ^‡^ *P* <0.0001; significantly different from all formula groups: ^§^ *P* <0.0001. Control = bovine milk-based, whey-predominant, alpha-lactalbumin-enriched term infant formula with 100% vegetable fat blend; HM = human milk; dw = dry weight; *sn*-2 = high *sn*-2 palmitate formula (Control formula modified to contain 60% vegetable fat blend and 40% high *sn*-2 palmitate fat blend); *sn*-2+OF = high *sn*-2 palmitate formula supplemented with oligofructose at 3.0 g/L.

**Figure 3:**
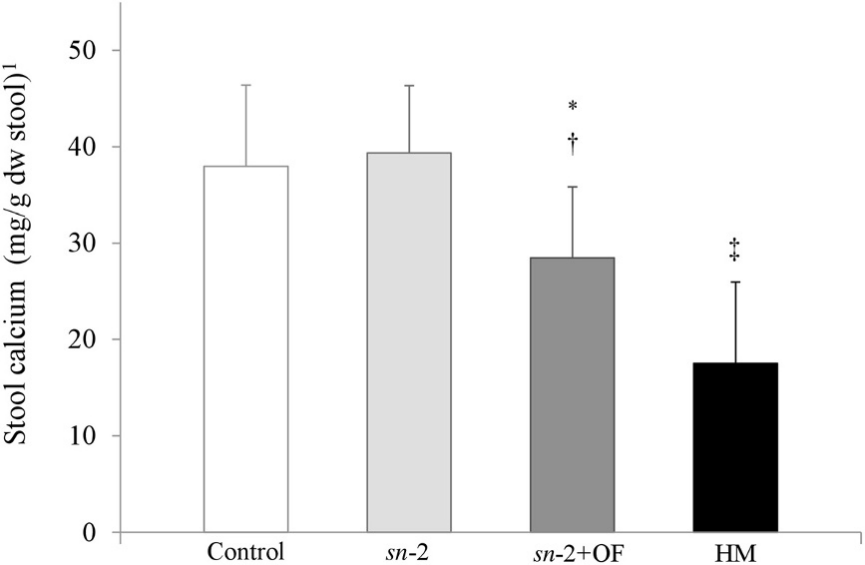
**Stool calcium (mg/g dry weight stool) at Day 28 according to feeding group.** ^*1*^Values are means ± SD. The overall formula-fed groups were analyzed by ANOVA followed by pairwise comparisons; the HM group was compared to each formula group using independent t-tests. Means (± SD) significantly different from Control: ^*^ *P* <0.0001; significantly different from *sn*-2: ^†^ *P* <0.0001; significantly different from all formula groups: ^‡^ *P* <0.0001. Control = bovine milk-based, whey-predominant, alpha-lactalbumin-enriched term infant formula with 100% vegetable fat blend; dw = dry weight; HM = human milk; *sn*-2 = high *sn*-2 palmitate formula (Control formula modified to contain 60% vegetable fat blend and 40% high *sn*-2 palmitate fat blend); *sn*-2+OF = high *sn*-2 palmitate formula supplemented with oligofructose at 3.0 g/L.

PA was the predominant fatty acid present in fecal fatty acid soaps in all groups (Table 3). The concentration of palmitate soaps in the *sn*-2 group (159.3 ± 42.0 mg/g dry weight stool) was significantly lower than the Control group (186.9 ± 48.8 mg/g; *P* =0.0028) and addition of OF to the formula resulted in a further reduction (113.3 ± 43.1 mg/g; *P* <0.0001) (Figure 2). The stool palmitate soaps of the HM-fed group were substantially lower than any of the formula groups (26.0 ± 26.2 mg/g; *P* <0.0001). Total fatty acid soap concentration in the *sn*-2 group was similar to the Control group while the *sn*-2+OF group had lower total soap fatty acids than either of the other two FF groups (*P* <0.0001, Table 3). The HM group had lower total fatty acid soap concentrations (63.5 ± 56.3 mg/g; *P* <0.0001) than any of the FF groups. Other saturated fatty acid soaps such as lauric (C12:0), myristic (C14:0), stearic (C18:0), oleic (C18:1) and linoleic (C18:2) were descriptively summarized (Table 3). Stool calcium concentration in the *sn*-2+OF group was significantly lower (28.5 ± 7.3 mg/g, *P* <0.0001) than in both the Control (38.0 ± 8.4 mg/g) and *sn*-2 groups (39.4 ± 7.0 mg/g) but was not significantly different between the Control group and the *sn*-2 group (Figure 3). Stool calcium in the HM-fed group was lower (17.6 ± 8.4 mg/g; *P* <0.0001 for each comparison) than all FF groups.

**Table 3:** **Stool fatty acid soaps (mg/g dry weight stool) at day 28**

Primary fatty acid soap endpoints ^***1***^	Feeding group				Statistically significant group differences ^***2***^
	Control	***sn*** -2	***sn*** -2+OF	HM	
	(n =46)	(n =51)	(n =49)	(n =48)	
C16:0 FA soap (palmitate)	186.9 ± 48.8	159.3 ± 42.0 ^*a*^	113.3 ± 43.1^*b, c*^	26.0 ± 26.2^*d*^	^*a*^ *P* =0.0028 vs*.* control
					^*b*^ *P* <0.0001 vs. control
Total FA soaps	272.8 ± 67.9	251.4 ± 65.1	175.6 ± 64.1^*b, c*^	63.5 ± 56.3^*d*^	^*c*^ *P* <0.0001 vs. *sn*-2
					^*d*^ *P* <0.0001 vs. all formulas
Secondary endpoints - individual FA soaps^*1, 3*^					
C12:0 FA soap (lauric)	4.6 ± 2.0	3.2 ± 1.4	1.6 ± 1.4	0.3 ± 0.4	
C14:0 FA soap (myristic)	13.7 ± 4.5	9.6 ± 3.0	6.0 ± 3.2	1.3 ± 1.5	
C18:0 FA soap (stearic)	37.8 ± 8.7	38.6 ± 9.8	31.4 ± 10.5	23.2 ± 20.5	
C18:1 FA soap (oleic)	20.0 ± 8.7	29.3 ± 9.9	15.5 ± 10.5	8.4 ± 8.8	
C18:2 FA soap (linoleic)	3.2 ± 1.6	3.4 ± 1.4	1.8 ± 1.7	1.6 ± 1.6	

^*1*^Mean ± SD.

^*2*^Based on analysis of variance followed by pairwise comparisons for formula groups, and independent t-tests for each of the formula groups vs. HM group.

^*3*^Descriptively summarized; no statistical analysis done.

Control = bovine milk-based, whey-predominant, alpha-lactalbumin-enriched term infant formula with 100% vegetable fat blend; FA = fatty acid; HM = human milk; *sn*-2 = high *sn*-2 palmitate formula (Control formula modified to contain 60% vegetable fat blend and 40% high *sn*-2 palmitate fat blend); *sn*-2+OF = high *sn*-2 palmitate formula supplemented with oligofructose at 3.0 g/L.

Statistical significance: ^*a:*^ *P* =0.0028 vs*.* control, ^*b:*^ *P* <0.0001 vs. control, ^*c:*^ *P* <0.0001 vs. *sn*-2 and ^*d:*^ *P* <0.0001 vs. all formulas.

### Stool consistency and frequency

**Figure 4:**
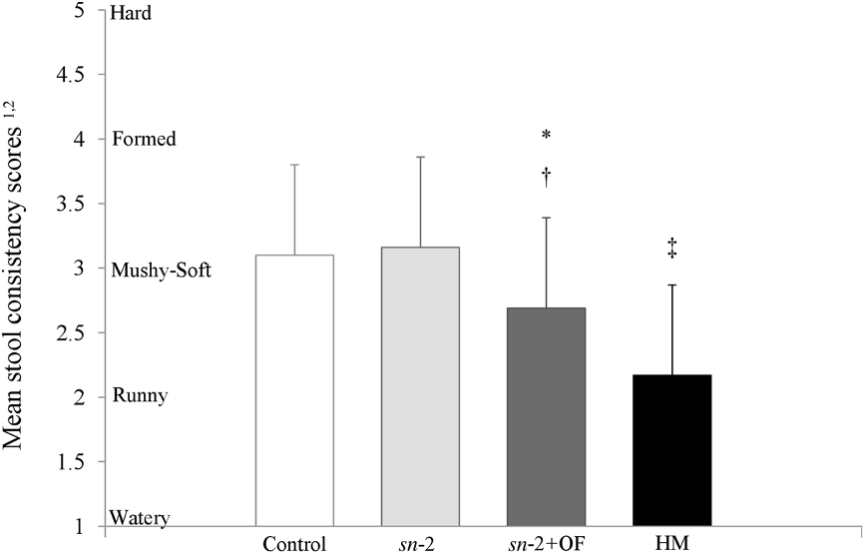
**Stool consistency scores at day 28 according to feeding group.** ^*1*^Individual stool consistency scores were determined using a five point scale for stool consistency (1 = watery, 2 = runny, 3 = mushy soft, 4 = formed, and 5 = hard). ^*2*^ Values are means (± SD). Means (± SD) significantly different from Control: ^*^ *P* <0.0001; significantly different from *sn*-2: ^†^ *P* <0.0001; significantly different from all formula groups: ^‡^ *P* <0.0001. Control = bovine milk-based, whey-predominant, alpha-lactalbumin-enriched term infant formula with 100% vegetable fat blend; HM = human milk; *sn*-2 = high *sn*-2 palmitate formula (Control formula modified to contain 60% vegetable fat blend and 40% high *sn*-2 palmitate fat blend); *sn*-2+OF = high *sn*-2 palmitate formula supplemented with oligofructose at 3.0 g/L.

The mean stool consistency is presented in Figure 4. Stool consistency was similar for the Control and *sn*-2 groups (3.1 ± 0.6 and 3.2 ± 0.4, respectively); scores represented a mean stool consistency that was mushy-soft. The stool consistency score of the *sn*-2+OF group (2.7 ± 0.5) was between runny (category 2) and mushy-soft (category 3), and was significantly lower than the other two FF groups (*P* <0.0001). The HM-fed group had a significantly lower stool consistency score than any of the FF groups (2.2 ± 0.5, *P* <0.0001), approaching the runny category. Hard stools were absent in the Control, *sn*-2+OF and HM-fed groups, and represented <0.4% of stools in the *sn*-2 group. Percent of formed stools were 23%, 19%, 2% and 0; percent mushy soft stools were 63%, 76%, 64%, and 23%; and percent runny stools were 13%, 4%, 34%, and 70% in the Control, *sn*-2, *sn*-2+OF, and HM-fed groups, respectively. While no FF infants had watery stools, 7% of the HM-fed infants did.

No differences in stool frequency were reported among the FF groups but stool frequency was lower (*P* <0.0001) in all FF groups compared with the HM-fed infants. The average number of stools per day at day 14 was 1.4, 1.2, 1.3, and 3.6, while at day 28 was 1.0, 0.9, 0.9, and 2.5, in the Control, *sn*-2, *sn*-2+OF, and HM-fed groups, respectively.

### GI tolerance

The GI Symptom Burden Index Scores were 18.9 ± 4.6, 19.2 ± 4.4, 18.7 ± 4.5, 18.5 ± 4.6 for Control, *sn*-2, *sn*-2+OF and HM-fed groups, respectively. These results indicate that the parent assessment of GI tolerance was similar across all groups and that the GI burden of each of the study feedings was low. Additionally, individual questions from the IGSQ showed no evidence of an increase in watery stools or increased gassiness with associated discomfort.

### Adverse events

The proportion of infants with physician-reported AEs that emerged during the study were 42.6% for the Control formula group, 46.4% for the *sn*-2 group, 35.8% for the *sn*-2+OF group and 40.0% for the HM-fed group. AEs were primarily distributed among GI disorders, skin/subcutaneous tissue disorders and infections. The percentages of physician-reported GI AEs were: 18.5%, Control group; 19.6%, *sn*-2 group; 7.5%, *sn*-2+OF group; and 9.1%, HM-fed group. Only three GI AEs were reported to be related to the study feedings, and all occurred in the Control group. One subject experienced hypomotility and another subject experienced both hard stools and constipation and discontinued from the study. No physician-reported, study-related GI adverse events occurred in either of the two experimental formula groups. The percentages of subjects with at least one SAE were 7.4% in the Control group, 5.4% in the *sn*-2 group, and 5.7% in the *sn*-2+OF group (the HM-fed group had no SAEs), and the investigators categorized these primarily as infections/infestations category. None of the SAEs were considered study-related.

### Hydration

All FF infants had urine osmolality and specific gravity values within the normal range (osmolality: 50–645 mOsmol/kg for subjects 0 to 30 days of age, and 50–1500 mOsmol/kg for subjects >30 days old at the time of urine collection; specific gravity: 1.003-1.035 for all infants). Due to difficulties in obtaining samples for all infants, the number of urine samples at day 28 for each group ranged from 31 to 38. Physicians reported no incidences of diarrhea in the Control, *sn*-2+OF or HM-fed groups and one case of diarrhea in the *sn*-2 group, which was not considered study-related.

### Anthropometry

Z-scores for weight, length and head circumference were near zero at baseline and days 14 and day 28. At day 28, the results were as follows: mean weight-for-age Z-scores were 0.015 ± 0.76, 0.110 ± 0.82, 0.073 ± 0.83, and −0.073 ± 0.74; length-for-age Z-scores were 0.015 ± 1.01, 0.027 ± 1.00, −0.029 ± 0.93, and 0.172 ± 0.82; and mean head circumference-for-age-Z-scores were −0.197 ± 0.78, 0.126 ± 0.98, −0.200 ± 0.88, and −0.129 ± 0.62, in Control, *sn*-2, *sn*-2+OF and HM-fed groups, respectively.

## Discussion

The present study demonstrates stool palmitate soaps were higher in infants fed a Control formula compared to the infants who received a formula containing high *sn*-2 palmitate structured lipid as part of the formula fat blend. These results are consistent with previous studies evaluating stool soaps [14, 15] or PA absorption [16] in term infants. In both the present study and in previous studies, the stool palmitate soap and calcium concentrations of breast fed infants are substantially lower than FF infants. This is most likely due to two characteristics of HM: the high concentration of *sn*-2 palmitate [2] and lower concentrations of calcium.

In previous studies evaluating higher *sn*-2 palmitate products, the experimental formulas contained approximately 45-66% of PA in the *sn*-2 position. [14–16]. Carnielli et al. compared a control formula to an ‘intermediate’ *sn*-2 palmitate formula (39% PA at *sn*-2) with a lipid blend similar to the *sn*-2 formulas used in the present study and a higher *sn*-2 palmitate formula (66% PA at *sn*-2, similar to HM) [16]. They found that feeding infants the intermediate *sn*-2 palmitate formula increased palmitate absorption but did not impact total fat or calcium absorption compared to the control formula group; whereas, feeding the high *sn*-2 palmitate formula (66% PA at *sn*-2) significantly increased absorption of all three parameters compared to the other 2 formula groups. The *sn*-2 formulas used in the present study consisted of a fat blend containing approximately 39% of PA at the *sn*-2 position. We found no reductions in fecal excretion of total fatty acid soaps and calcium in infants fed the formula containing *sn*-2 only. These observations suggest that the formation of fatty acid soaps is in part dependent on the concentration of the *sn*-2 palmitate in the structured lipid provided.

The present study demonstrates that soap formation may also be influenced by factors independent of triglyceride structure, such as the presence of the prebiotic OF. This prebiotic is a non-digestible oligosaccharide which is transported intact to the colon [17]. Here it is fermented by bacteria that are beneficial to the infant such as bifidobacteria and lactobacilli to generate a series of short chain organic fatty acids, such as acetate, propionate and butyrate as well as lactate [9, 18] and may impact intestinal pH [19]. Feeding prebiotics to infants results in a reduction in fecal pH compared with infants receiving control formula [20, 21]; this lower colonic pH may lead to increased solubilization of minerals and increased calcium absorption [8]. Prebiotic fibers also exert a trophic effect on epithelial cells throughout the GI tract to improve mineral absorption, as demonstrated in animals [22]. Several studies have demonstrated an increase in calcium absorption and bone mineralization following ingestion of prebiotic mixtures in pre-adolescents and adolescents [9, 23]. Stable isotope studies demonstrate that this effect is due to increased colonic calcium uptake [9]. In the present study, infants fed the *sn*-2+OF formula had lower stool total fatty acid soaps and calcium concentrations than infants fed the Control or *sn*-2 formulas, suggesting that a reduction in colonic pH may increase the solubility of calcium in the colon, improving retention. A metabolic balance study would confirm whether the reduced fecal calcium excretion observed in this study corresponds to improved intestinal calcium retention and absorption, and hence the availability of more calcium for bone mineralization.

Stool consistency differs between breastfed and FF infants, with breastfed infants having softer stools [5, 10]. Quinlan et al. reported that fatty acid soaps and calcium were the dominant factors related to stool hardness in both FF and breastfed infants [5]. Besides the presence of fatty acid soaps and calcium, other factors may impact stool consistency including consumption of prebiotics such as oligosaccharides, which occur naturally in breast milk [24]. Consumption of oligosaccharides has been shown to soften stools in infants [25, 26]. The mechanism for OF-mediated stool softening differs from that of high *sn*-2 palmitate structured lipids, although the exact mechanism is unclear. It is known that OF serves as a substrate for select colonic bacteria to increase microbial mass, increase stool bulk [27] and improve stool consistency [26, 28]. In the present study, infants fed *sn*-2+OF formula had significantly softer stools, and lower total soaps, palmitate soaps and calcium than infants fed either *sn*-2 or Control formula. Although there was a difference in the palmitate soaps between the Control and *sn*-2 groups, stool softness, total fatty acid soap, and stool calcium concentrations did not differ between them, suggesting that other factors besides a reduction in palmitate soaps contributes to stool softness.

GI tolerance determined using the IGSQ survey and physician reported GI adverse events were similar in all groups. Some studies have reported an increased incidence of softer, looser or watery stools in formulas supplemented with prebiotics compared to control formulas [6, 18, 29]. A formula containing both a structured lipid (high *sn*-2 PA) and a mixture of galacto-oligosaccharide and fructo-oligosaccharide (8 g/L) resulted in a higher proportion of watery and runny stools than a control formula [29]. The EU raised concerns about water balance and the potential for dehydration of infants receiving such formulas [30]. However, Closa-Monasterolo et al. reported normal water balance when infants received a formula containing 8 g/L of an inulin/OF mix [31]. The current study did not find an increase in the number of watery stools in the *sn*-2+OF group compared to the other 2 formula groups. In addition, no cases of diarrhea were reported in the *sn*-2+OF group and only one case in the *sn*-2 group. These results demonstrate that the formulas evaluated in this study do not negatively impact water balance of term infants.

## Conclusions

This study demonstrates the interaction of multiple dietary components to provide beneficial outcomes on stool composition and consistency in term infants. Feeding IF containing *sn*-2 palmitate and OF results in reduced palmitate soaps, total fatty acid soaps, and calcium in stool and is well tolerated. This combination of ingredients also promotes the formation of softer stools which are of intermediate consistency between those of HM-fed infants and those of infants fed standard or *sn*-2 only enriched formulas.

## Electronic supplementary material

Additional file 1: **Statistical Methods.** (DOCX 24 KB)

## Abbreviations

AE: Adverse event
Control: Bovine milk-based, whey-predominant, alpha-lactalbumin-enriched term infant formula with 100% vegetable fat blend
dw: Dry weight
FF: Formula fed
GI: Gastrointestinal
HM: Human milk
IF: Infant formula
IGSQ: Infant Gastrointestinal Symptom Questionnaire
OF: Oligofructose
PA: Palmitic acid
SAE: Serious adverse event
*sn-*2: High *sn*-2 palmitate formula (Control formula modified to contain 60% vegetable fat blend and 40% high *sn*-2 palmitate fat blend)
sn-2+OF: High *sn*-2 palmitate formula supplemented with oligofructose at 3.0 g/L.
