# Supplementary material for: Stool fatty acid soaps, stool consistency and gastrointestinal tolerance in term infants fed infant formulas containing high sn-2 palmitate with or without oligofructose: a double-blind, randomized clinical trial
Source: Nutr J. 2014 Nov 5;13:105. doi: 10.1186/1475-2891-13-105 (PMC4273321; doi:10.1186/1475-2891-13-105)
Supplement: Supplementary file 1 — Additional file 1: Statistical Methods. (DOCX 24 KB) [file 12937_2014_846_MOESM1_ESM.docx]

Title

Stool fatty acid soaps, stool consistency and gastrointestinal tolerance in term infants fed infant formulas containing high sn-2 palmitate with or without oligofructose: a double-blind, randomized clinical trial

Authors

Joyce Nowacki**,** Hung-Chang Lee, Reyin Lien, Shao-Wen Cheng, Sung-Tse Li, ManjiangYao, Robert Northington, Ingrid Jan and Gisella Mutungi

**Additional Details on Statistical Methods**

Sample Size Calculations

The sample size calculation was based on the primary endpoint (stool fatty acid soaps) and also on the key secondary endpoint (stool consistency). Specifically, the stool fatty acid soap calculation was based on mean fatty acid soaps and was determined from data in infants fed control and high sn-2 formula [[1](#_ENREF_1), [2](#_ENREF_2)] as well as the within-group SD value derived from a study conducted by the sponsor in a similar ethnic population of Chinese infants fed standard term IF (unpublished data). A sample size of 23 infants per group was required to provide 90% power to declare a mean difference of 7% in fatty acid soaps (SD of 7%) between the control and *sn*-2 group to be statistically significant at the 0.05 level. Regarding stool consistency, based on the SD value obtained from a prior study conducted by the sponsor (unpublished data), and the mean score difference found from the study by Kennedy et al. [13] (1999), a minimum of 40 infants per group was required to provide 90% power to declare a mean difference between groups of 0.3 (SD of 0.4) points in the 5-point stool consistency rating scale to be statistically significant at the 0.05 level. Taken together, a sample size of at least 40 infants per group was found to provide sufficient power to detect differences in stool fatty acid soaps and stool consistency. To account for subjects lost from analysis, 55 infants per formula group (a total of 165 formula fed [FF] infants) were to be enrolled to ensure at least 40 infants per formula group (a total of 120) were available for analysis. In addition, approximately 55 HM-fed infants were also to be enrolled. Therefore, the total sample size for this study was approximately 220 infants.

Multiple Comparisons

With regard to the endpoints, for multiple comparison purposes, palmitate soap was considered the most important component of the stool composition endpoints. Stool consistency was considered the most important of the stool characteristics endpoints, and was a key secondary endpoint.

All of the pairwise comparisons were done at α = 0.05. A stepwise process was followed to control for multiple endpoints. The first comparison was *sn*-2+OF vs. control for the palmitate soap endpoint. If this was found to be significant at *P*<0.05, then sn-2+OF was to be compared to control formula for stool consistency.

In addition, if *sn*-2+OF vs. control was significant at *P* < 0.05 for palmitate soap, then *sn*-2 was to be compared to control for this same endpoint. If this comparison was found to be significant at 0.05, then *sn*-2 and control were to be compared for stool consistency. Unless otherwise specified, all remaining comparisons of endpoints were not to be adjusted for multiplicity.

**References**

1. Kennedy K, Fewtrell MS, Morley R, Abbott R, Quinlan PT, Wells JC, Bindels JG, Lucas A: **Double-blind, randomized trial of a synthetic triacylglycerol in formula-fed term infants: effects on stool biochemistry, stool characteristics, and bone mineralization.** *Am J Clin Nutr* 1999, **70:**920-927.

2. Lucas A, Quinlan P, Abrams S, Ryan S, Meah S, Lucas PJ: **Randomised controlled trial of a synthetic triglyceride milk formula for preterm infants.** *Arch Dis Child Fetal Neonatal Ed* 1997, **77:**F178-184.
